# Extracellular Vesicles as Therapeutic Tools for the Treatment of Chronic Wounds

**DOI:** 10.3390/pharmaceutics13101543

**Published:** 2021-09-23

**Authors:** Eric R. Bray, Alisha R. Oropallo, Daniel A. Grande, Robert S. Kirsner, Evangelos V. Badiavas

**Affiliations:** 1 Phillip Frost Department of Dermatology and Cutaneous Surgery, University of Miami Miller School of Medicine, Miami, FL 33136, USA; erbray@med.miami.edu (E.R.B.); rkirsner@med.miami.edu (R.S.K.); 2 Interdisciplinary Stem Cell Institute, University of Miami Miller School of Medicine, Miami, FL 33136, USA; 3 Comprehensive Wound Healing Center and Hyperbarics, Department of Vascular Surgery, Donald and Barbara Zucker School of Medicine, Hofstra/Northwell Health, Hempstead, NY 11549, USA; Aoropallo@northwell.edu (A.R.O.); DGrande@northwell.edu (D.A.G.); 4 Feinstein Institutes for Medical Research, Northwell Health, Manhasset, NY 11030, USA; 5 Department of Orthopedic Surgery, Long Island Jewish Medical Center, Northwell Health, New Hyde Park, NY 11040, USA

**Keywords:** chronic wound, extracellular vesicles, mesenchymal stem cell, wound healing, drug delivery, biomaterial

## Abstract

Chronic wounds develop when the orderly process of cutaneous wound healing is delayed or disrupted. Development of a chronic wound is associated with significant morbidity and financial burden to the individual and health-care system. Therefore, new therapeutic modalities are needed to address this serious condition. Mesenchymal stem cells (MSCs) promote skin repair, but their clinical use has been limited due to technical challenges. Extracellular vesicles (EVs) are particles released by cells that carry bioactive molecules (lipids, proteins, and nucleic acids) and regulate intercellular communication. EVs (exosomes, microvesicles, and apoptotic bodies) mediate key therapeutic effects of MSCs. In this review we examine the experimental data establishing a role for EVs in wound healing. Then, we explore techniques for designing EVs to function as a targeted drug delivery system and how EVs can be incorporated into biomaterials to produce a personalized wound dressing. Finally, we discuss the status of clinically deploying EVs as a therapeutic agent in wound care.

## 1. Introduction

Cutaneous wound healing is complex, consisting of overlapping processes: hemostasis/coagulation, inflammation, proliferation, and remodeling [1]. This requires intercellular communication among resident cells and entering immune cells through soluble, membrane-bound, and extracellular matrix (ECM) molecules [1,2]. Wounds that fail to heal in a timely process are called chronic wounds [3]. A 2004 meta-analysis found that in the United States skin ulcers and wounds were associated with USD 9.7 billion in annual direct medical costs [4]. For patients, chronic wounds cause pain, loss of productivity, a profound impact on quality of life, and increased mortality [4,5,6]. Risk factors for the development of chronic wounds include advanced age, diabetes mellitus with associated peripheral vascular disease and peripheral neuropathy, as well as chronic kidney disease and immobility [5,7]. The societal burden of chronic wounds will increase as the population ages and the prevalence of co-morbid chronic conditions continues to rise. Current advanced therapies, including topical application of growth factors [8], extracellular matrix products [9], and skin substitutes [10], are not always effective [11]. Therefore, it is imperative that cutting-edge therapeutics be identified to treat chronic wounds. 

The goals of this review are as follows: (1) briefly discuss the benefits and limitations of mesenchymal stem cell (MSC) therapy for treating chronic wounds and how MSC derived extracellular vesicles (EVs) overcome many of these limitations; (2) examine in detail the effects of MSC-EVs on each stage of the wound healing process; (3) explore techniques for modifying MSC-EVs; and (4) highlight the safety and regulatory aspects of using MSC-EVs as a therapeutic agent in wound care. We provide new perspectives regarding how MSC-EVs can be engineered to further enhance their therapeutic efficacy by synthesizing our understanding of chronic wound pathophysiology and the mechanism of action of MSC-EVs.

## 2. From Mesenchymal Stem Cells to Extracellular Vesicles

### 2.1. Lessons Learned from Mesenchymal Stem Cells

Stem cells provide promise in the field of regenerative medicine. They possess the capacity for self-renewal and differentiation into multiple cell types. Ideally, stem cells could improve the quantity and quality of healing by accelerating the rate of wound healing, transforming non-healing wounds into actively healing wounds, reducing scarring, and regenerating skin appendages [12]. Multiple stem cell sources exist, with distinct advantages and disadvantages to each, and are reviewed elsewhere [12,13]. 

Amongst the stem cell populations, mesenchymal stem cells (MSCs) have received the most attention in wound healing research [14]. MSCs are distributed throughout the body and are believed to play important roles in tissue homeostasis, repair, and regeneration [15]. The primary sources of MSCs for clinical research are the bone marrow (BM-MSCs), adipose tissue (AD-MSCs), and umbilical cord (UC-MSCs) [16]. A particular advantage of MSCs is they are relatively easy to harvest from adult tissue or tissue that would be otherwise discarded, limiting ethical concerns regarding their use. MSCs are defined as plastic adherent cells that express CD73, CD90, and CD105, while not expressing hematopoietic lineage markers CD14, CD34, CD45, and HLA-DR, and having the capacity to differentiate into osteoblasts, chondroblasts, and lipoblasts [17]. 

Given their immune-privileged/immune-modulatory nature, BM-MSCs can be used in unmatched recipients without the need for typing [18,19]. The clinical utility of BM-MSCs is enhanced by the ability to use allogeneic cells. MSCs exert an array of beneficial effects through each phase of the wound healing process [20,21]. Clinical trials have demonstrated that autologous and allogeneic MSC therapy aids in chronic wound closure [22,23,24,25,26,27,28,29,30]. To date, there have been well over 1000 clinical trials using MSCs [31,32]. Thus far, no significant adverse events have been reported related to the administration of these cells in humans [33]. The substantial therapeutic benefits offered by MSCs however do not exist without potential drawbacks. The need to maintain cell viability imposes technical challenges on cell generation, storage, transportation, and clinical administration. Murine studies have raised concerns for pulmonary vascular congestion due to cells accumulating in the pulmonary microvasculature [34] and ectopic tissue formation [35]. While MSCs are considered less carcinogenic than other stem cell sources, this important caveat warrants consideration. Critically, no evidence of tumor formation has been reported to date [32,36], but continued surveillance is warranted. Concern for acquiring chromosome abnormalities may limit the rate at which and the number of passages cells can be expanded in vitro [36]. Genetic modification of MSCs may allow for more precise targeting of specific biologic problems and increase therapeutic efficacy [13]. Importantly, the tumorgenicity of any stable genetic alteration needs to be considered prior to the delivery of modified cells to a patient. 

Despite the potential for MSCs to differentiate into multiple cell lineages, only a small number of transplanted MSCs are incorporated into repaired tissue [37,38]. Instead, MSCs are envisioned as a source of growth factors that promote tissue repair and potent immune modulators [13,20,39,40]. For example, BM-MSC conditioned media (CM) can accelerate wound healing and promote the recruitment of macrophages and endothelial cells [41]. Furthermore, it is recognized that many of the beneficial effects of MSCs in cutaneous wound healing are mediated through the secretion of extracellular vesicles [42,43,44,45,46,47]. 

### 2.2. Extracellular Vesicles

**Figure 1:**
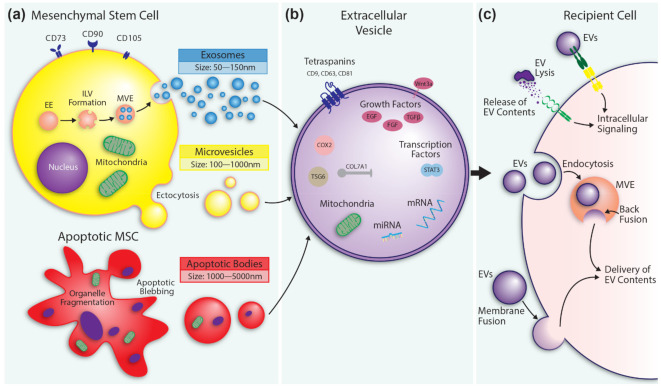
Mesenchymal stem cell (MSC) extracellular vesicle (EV) formation and messaging. (**a**) Exosome formation begins with membrane endocytosis of the early endosome (EE) to form intraluminal vesicles (ILV). ILVs are contained within a multivesicular endosome (MVE). Exosomes are released following fusion of the MVE with the plasma membrane. Microvesicles are released by ectocytosis and budding from the plasma membrane. Apoptotic bodies form from cells undergoing apoptosis and may contain fragmented organelles. (**b**) Depiction of select EV contents that contribute to wound healing. Additional EV contents are discussed in the main text and Table 1. (**c**) Released EVs interact with a recipient cell through membrane receptors thereby initiating intracellular signaling. EVs can also deliver their cargo to the recipient cell following endocytosis and back fusion within an MVE or by direct fusion with the plasma membrane. Lysis of EVs in the extracellular space releases contents that then act on the recipient cells.

Extracellular vesicles (EVs) are lipid bilayer vesicles that can be secreted by all cell types [48]. The term “extracellular vesicle” is generic, referring to any lipid bilayer secreted vesicle. EVs are a heterogeneous population consisting of exosomes, microvesicles, and apoptotic bodies (Figure 1a). They differ in size, morphology, density, cargo, biogenesis, and biologic activity. Given the heterogeneity and challenges distinctly classifying these populations, we will use the term EV throughout the paper to refer to all classes of vesicle. 

Exosomes are secreted intraluminal vesicles (ILV). Inward budding of the endosomal membrane results in the formation of ILVs in a multivesicular endosome (MVE). Exosomes are released by fusion of the MVE with the plasma membrane [49]. Microvesicles form as outward protrusions (ectocytosis) of the plasma membrane [50]. A cell undergoing apoptosis breaks down its cellular components and organelles and packages them into apoptotic bodies [51,52]. 

EVs carry an array of bioactive molecules that regulate intercellular communication [53,54] and promote wound healing (Figure 1b) [42,43,44,45,46,47]. Additionally, EVs can deliver functional mitochondria to recipient cells [55]. The content of EVs is highly heterogenous and is influenced by the cell of origin, microenvironment, active signals within a cell, and isolation procedures [56,57].

Functional messenger RNAs (mRNA) are transferred between cells by EVs [58]. MicroRNAs (miRNA) are selectively enriched in EVs and regulate gene expression in recipient cells [59,60,61]. Further studies have identified that EVs contain genomic DNA, mitochondrial DNA (mtDNA), ribosomal RNA (rRNA), transfer RNA (tRNA), long noncoding RNA (lncRNA), circular RNA (circRNA), and picoRNA (piRNA) [56,62,63,64,65]. 

Tetraspanin proteins are enriched in the membrane of EVs and regulate membrane structure, trafficking, and fusion with recipient cells [66]. EVs also contain adhesion molecules, ESCRT proteins, heat-shock proteins, cytoskeletal proteins, enzymes, and proteins involved in antigen presentation, membrane trafficking, and signal transduction [48,53]. Additionally, proteins such as Wnt3a are associated with the exterior of EVs [67]. 

EV membranes are enriched with cholesterol, sphingomyelin, phosphatidic acid, and ceramides. EVs have also been shown to transport bioactive lipids such as prostaglandins, leukotrienes, and fatty acids [68]. Online databases have been created to catalogue EV cargos, namely EXOCARTA (exocarta.org, accessed on 16 September 2021) and Vesiclepedia 2019 (microvesicles.org, accessed on 16 September 2021) [69,70]. 

The biodistribution of EVs is dependent on their cell of origin and expression of surface molecules [56]. Their half-life in circulation ranges from minutes to hours [71,72,73]. Clearance by the reticuloendothelial system can be prolonged by the expression of anti-phagocytic surface proteins: CD47, THBS-1, and SIRPα [74]. Upon reaching a target EVs may bind surface receptors initiating intracellular signaling or deliver their contents by endocytosis or fusion with the plasma membrane (Figure 1c) [48]. Additionally, the lysis of EVs releases their cargo into the extracellular space [75,76]. 

## 3. Role for MSC Extracellular Vesicles in Wound Healing

There is accumulating pre-clinical evidence that MSC-EVs are beneficial in cutaneous wound healing (Table 1). In this section, we will discuss how MSC-EVs can influence key components of the wound healing process.

**Table 1:** Studies that evaluated an in vivo role for MSC-EVs in wound healing.

Study	EV Source	Model	Findings
Fang et al. 2016 [77]	Human UC-MSC	Mouse skin wound -Local injection	EVs reduced scar formation and myofibroblast accumulation.
		In vitro dermal fibroblasts	EVs suppressed TGF-β induced myofibroblast formation. EVs were enriched in miR-21, miR-23a, miR-125b, and miR-145. miRNA delivery reduced TGF-β/SMAD2 signaling in fibroblasts.
Hu et al. 2016 [78]	Human AD-MSC	Mouse skin wound -Local injection	EVs improved rate of wound healing, increased Col1 and Col3 mRNA on Day 3 and Day 5 post wounding, and decreased Col1 and Col3 mRNA on Days 7 and 14.
		Mouse skin wound -Intravenous injection	EVs migrated to wound site (Days 5–14) and spleen and promoted wound healing.
		In vitro fibroblasts	EVs promoted fibroblast proliferation and migration, increased mRNA for N-cadherin, COL1, COL3, and elastin.
Zhang et al. 2018 [79]	Human AD-MSC	Mouse skin wound -Local injection	EVs improved rate of wound healing, decreased scar size, and neoangiogenesis.
		In vitro fibroblasts	EVs promoted fibroblast proliferation and migration, and increased mRNA for COL1, COL3, MMP1, FGF2, and TGF-β1. Fibroblasts had increased p-AKT. Application of PI3K/AKT inhibitor Ly294002 abrogated the EV-induced effects on fibroblasts.
He et al. 2019 [80]	Human BM-MSC	Mouse skin wound -Intravenous injection	EVs promoted wound healing and polarization of macrophages to M2 phenotype.
		In vitro human monocytes/macrophages	EVs promoted M2 macrophage polarization in part through transfer of miR-223.
Ren et al. 2019 [81]	Human AD-MSC	Mouse skin wound -Local injection	EVs accelerated wound healing, re-epithelialization, collagen deposition, and neovascularization.
		In vitro fibroblasts, keratinocytes (HaCaT), and endothelial cells (HUVEC)	EVs promoted proliferation and migration, and stimulated AKT and ERK signaling.
Cheng et al. 2020 [82]	Human UC-MSC	Mouse skin wound -Local injection	EVs accelerated re-epithelialization and promoted collagen fiber maturation.
		In vitro dermal fibroblasts and keratinocytes (HaCaT)	EVs promoted proliferation and migration. The effect was blocked by miR-27b inhibitor. Proposed miR-27b acts by suppressing ITCH, thereby activating JUNB/IRE1α.
Jiang et al. 2020 [83]	Human BM-MSC	Mouse skin wound -Local injection	EVs from MSCs with TSG-6 overexpression (TSG-6-EVs) and knock-down (TSG-6-KD-EVs). EVs reduced scar formation, reduced production of TGF-β1, Collagen I and III, and αSMA protein, and suppressed SMAD2/3 signaling. TSG-6-EVs enhanced the effect of EVs, the effect was lost in TSG-6-KD-EVs, and when TSG-6 neutralizing antibodies were present.
Liu et al. 2020 [84]	Mouse BM-MSC	Mouse skin wound -Topical in Pluronic F127 hydrogel	Topical EVs accelerated wound healing, limited inflammatory infiltrate, and decreased scar size.
		In vitro mouse macrophages	EVs polarized macrophages towards M2 phenotype. Conditioned media from EV treated macrophages promoted fibroblast proliferation and migration.
Qiu et al. 2020 [85]	Mouse BM-MSC	Mouse skin wound -Local injection	EVs from MSCs treated with EVs from neonatal serum and adult serum. MSC-EVs accelerated wound healing and promoted neoangiogenesis. Neonatal serum stimulated MSC-EVs showed more robust effect.
		In vitro endothelial cells (HUVECs)	MSC-EVs promoted HUVEC proliferation, migration, and tube formation, and increased p-AKT and p-eNOS. Neonatal serum stimulated MSC-EVs showed more robust effect.
Zhang et al. 2020 [86]	Human AD-MSC	Mouse skin wound -Local injection	EVs promoted mouse wound healing, proposed to occur in AKT/HIF-1α dependent fashion.
		In vitro HaCaT Keratinocytes	EVs promoted HaCaT keratinocyte proliferation.
Zhao et al. 2020 [87]	Human UC-MSC	Mouse skin wound -Local injection	EVs enhanced re-epithelialization and neoangiogenesis.
		In vitro keratinocytes (HaCaT)	EVs stimulated keratinocyte proliferation, migration, and suppressed ROS induced apoptosis. Proposed effect was through suppression of AIF nuclear translocation and PARP-1 activation.
Li et al. 2021 [88]	Human AD-MSC	In vitro human hypertrophic scar fibroblasts	EVs decreased collagen deposition, trans-differentiation of fibroblasts-to-myofibroblasts, and formation of hypertrophic scar. EVs were noted to express miR-192-5p, which can suppress IL-17RA/SMAD axis.
Diabetic wounds:			
Wang et al. 2019 [89]	Mouse AD-MSC	Mouse diabetic wound -Topical in complex hydrogel (Pluronic F127, oxidative hyaluronic acid, and Poly-l-lysine)	EVs improved wound healing and neovascularization. The effect was improved when EVs were loaded in complex hydrogel.
Li et al. 2020 [90]	Mouse BM-MSC	Mouse diabetic wound -Local injection	EVs from MSCs overexpressing lncRNA H19 (H19-EVs). Only H19-EVs promoted wound healing, decreased inflammatory infiltrate, and increased granulation tissue formation.
		In vitro human fibroblasts from diabetic foot ulcers and health control	H19-EVs reduced miR-152-3p expression in fibroblasts from diabetics and increased PTEN expression.
Shi et al. 2020 [91]	Mouse AD-MSC	Mouse diabetic wound -Local injection	EVs accelerated wound healing, increased angiogenesis, suppressed apoptosis, and increased autophagy markers SIRT1 and LC3. The effects were further enhanced with EVs from mmu_circ_0000250 overexpressing MSCs.
		In vitro endothelial cells (HUVECs)	EVs promoted HUVEC survival under high glucose conditions and increased autophagy. This was enhanced by loading with mmu_circ_0000250, which was shown to increase SIRT1 mediated autophagy.
Yang et al. 2020 [92]	Human UC-MSC	Mouse diabetic wound -Topical in Pluronic F127 hydrogel	EVs accelerated wound healing and angiogenesis, increased expression of VEGF and TGF-β1.
Pomatto et al. 2021 [93]	Human BM-MSC AD-MSC	Mouse diabetic wound -Topical in carboxymethylcellulose	AD-MSC-EVs, but not BM-MSC-EVs, promoted the rate of wound healing. Comparative in vivo analysis of scar and angiogenesis was not performed.
		In vitro fibroblasts, keratinocytes, and endothelial cells	BM-MSC-EVs promoted proliferation of keratinocytes and endothelial cells, and promoted viability of fibroblasts, keratinocytes, and endothelial cells. AD-MSC-EVs promoted only the proliferation of endothelial cells. Protein and miRNA analysis indicated BM-MSC-EVs are enriched for proliferative factors, whereas AD-MSC-EVs are enriched in proangiogenic factors.
Ti et al. 2015 [94]	Human UC-MSC	Rat diabetic wound -Local injection	EVs from LPS preconditioned MSCs (LPS Pre-EVs) decreased inflammatory cell infiltration and polarized macrophages towards M2.
		In vitro human monocytes (THP-1)	LPS Pre-EVs induced M2 polarization. EVs transferred Let-7b, reducing TLR-4 expression and NF-kB activation.
Li et al. 2018 [95]	Human AD-MSC	Rat diabetic wound	EVs from MSCs overexpressing NRF2 (NRF2-EVs).Endothelial progenitor cells (EPC) + NRF2-EVs promoted wound healing better than EPC + AD-MSC-EVs, and both were better than EPC alone or control.
		In vitro human epithelial progenitor cells (EPC)	EVs decreased EPC senescence under high glucose conditions. NRF2-EVs inhibited inflammatory cytokines and ROS.
Ding et al. 2019 [96]	Human BM-MSC	Rat diabetic wound -Local injection	EVs from deferoxamine stimulated MSCs (DFO-EVs). EVs promoted wound healing and neoangiogenesis, and DFO-EVs were more effective.
		In vitro endothelial cells (HUVECs)	DFO-EVs were more potent stimulators of HUVEC proliferation and tube formation than EVs. DFO-EVs proposed to transfer miR-126 to HUVECs, which suppresses PTEN, and thereby activates AKT signaling.
Liu et al. 2020 [97]	Human BM-MSC	Rat diabetic wound -Local injection	EVs from MSCs treated with melatonin (MT-EVs). EVs promoted wound closure, Collagen I and III expression, and M2 macrophage polarization; MT-EVs enhanced the effect of EVs.
		In vitro mouse macrophages (RAW264.7)	MT-EVs were more potent than EVs at polarizing macrophages to M2 phenotype.
Yu et al. 2020 [98]	Human BM-MSC	Rat diabetic wound -Local injection	EVs from MSCs treated with atorvastatin (ATV-EVs). EVs promoted wound healing and angiogenesis. ATV-EVs were more effective.
		In vitro endothelial cells (HUVECs)	EVs promoted proliferation, migration, and tube formation, increased VEGF secretion, and activated AKT/eNOS signaling. ATV-EVs produce a larger magnitude effect compared to standard EVs. ATV-EVs proposed to work by upregulating miR-221-3p in endothelial cells.
Burn wounds:			
Shafei et al. 2020 [99]	Human AD-MSC	Mouse burn wound -Topical in alginate hydrogel	EVs accelerated wound closure, increased epithelial thickness, collagen deposition, and neovascularization.
Zhang et al. 2015 [100]	Human iPSC-MSC	Rat burn wound -Local injection	EVs accelerated re-epithelialization, reduced scar width, promoted collagen maturation, and stimulated neoangiogenesis. Effects depended on EV transfer of Wnt4.
		In vitro fibroblasts and endothelial cells (HUVECs)	EVs stimulated proliferation and migration, stimulated Collagen I and III, and elastin secretion, and promoted tube formation.
Li et al. 2016 [101]	Human UC-MSC	Rat burn wound -Intravenous injection	EVs reduce inflammation following burn wounds. EVs transfer miR-181c and reduce TLR4 signaling.
		In vitro mouse macrophages (RAW264.7)	EVs suppress LPS induced macrophage inflammation.

### 3.1. Inflammation

Wound healing is initiated immediately following tissue injury. Vascular injury and serum-derived factors promote clot formation and hemostasis at the site of trauma. There is rapid local production of pro-inflammatory cytokines (e.g., IL-1, IL-2, IL-6, IL-8, TNF-α, interferons (IFNs), and prostaglandins) and growth factors (TGF-β, EGF, PDGF, and FGF) [102]. These factors promote the migration of inflammatory cells into the wound environment. 

**Figure 2:**
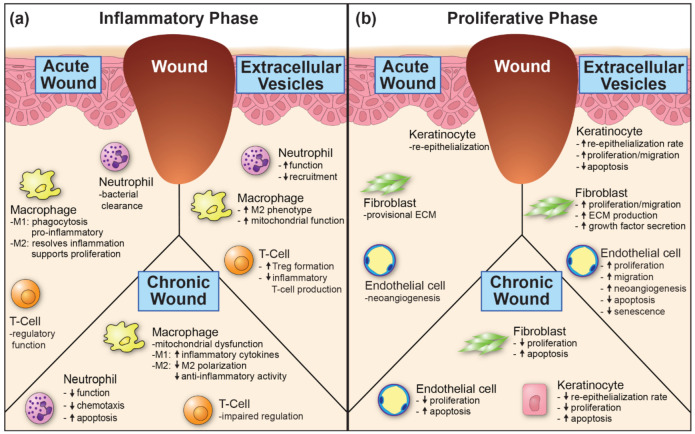
Diagram of key cellular components of the (**a**) inflammatory phase and (**b**) proliferative phase of wound healing. Panels depict cell function in the acute wound setting (left), changes that occur in chronic wounds (bottom), and how extracellular vesicles (EVs) can influence cell function (right). Arrows depict how a stated cell function is increased or decreased in the setting of chronic wounds relative to acute wounds, and then how EVs can increase or decrease the function relative to chronic wounds. ECM—extracellular matrix.

Neutrophils are the first inflammatory cell recruited. They are critical for controlling the invasion of bacteria through the compromised cutaneous barrier (Figure 2). Neutrophils remove bacterial seeding by phagocytosis, producing reactive oxygen species (ROS), and releasing cytotoxic molecules [103]. The molecules released by neutrophils also promote the breakdown and clearance of cellular debris. Dysfunctional neutrophils may contribute to the formation of chronic wounds. Neutrophils from patients with diabetes mellitus (DM) have an impaired respiratory burst, a weaker chemotactic response, and are more prone to apoptosis [104,105]. The function of genetically defective neutrophils can be improved with EV treatment [106]. In this context EVs may be able to restore impaired neutrophil function associated with diabetes, potentially resulting in the recruitment of fewer neutrophils during the wound healing process. Additionally, excessive neutrophil recruitment is also found in chronic wounds [107]. MSC-EVs can inhibit the infiltration of neutrophils into corneal wounds [108]. It remains to be determined if inhibition of neutrophil infiltration is due to EVs acting on neutrophils, or if it is a response to reduced inflammatory cytokine secretion into the wound environment. The effects of MSC-EVs on neutrophils, and other cells to be discussed, may seem contradictory. But it is important to consider that like MSCs, MSC-EVs are likely working to restore tissue homeostasis and do not act on any one cell in isolation. 

Macrophages play a dual role in wound healing. Murine studies suggest that macrophages initially assume the M1 pro-inflammatory phenotype. M1 macrophages release pro-inflammatory cytokines and phagocytose bacteria, ECM, and apoptotic cells. After damaged tissues have been cleared, the wound progresses into the proliferative phase. For this transition to appropriately occur, macrophages must also transition from their pro-inflammatory M1 phenotype to their anti-inflammatory M2 phenotype. M2 macrophages act to resolve inflammation through the secretion of anti-inflammatory cytokines such as IL-10 and IL-1RA. M2 polarized macrophages are a key source of growth factors (EGF, TGF-β, IGF-1) that regulate the proliferative phase and promote fibrosis. Importantly, inappropriate macrophage activation has been linked to scarring and the development of chronic wounds [109]. 

Numerous studies have investigated the influence of EVs on macrophages, reviewed elsewhere [110]. In the context of wound healing and tissue repair, EVs promote polarization to the M2 macrophage phenotype [80,84,94,97,101]. Acquisition of the M2 phenotype is associated with reduced expression of pro-inflammatory cytokines (TNF-α, IL-1, IFN-γ) and increased expression of anti-inflammatory cytokines (IL-4, IL-10). He et al. found intravenously (IV) injected BM-MSCs home to the wound site, promote M2 macrophage polarization, and improve wound healing [80]. These BM-MSCs failed to promote wound healing if macrophages were depleted, or if the BM-MSCs were unable to secrete EVs. Finally, they proposed that the effect was due to the transfer of miR-223 to macrophages [80]. EVs may also promote M2 polarization through the transfer of miR-let7 [94,111], miR-181c [101], and miR-182 [112]. 

Apoptosis of transplanted MSCs can inhibit inflammation and hypertrophic scarring [113]. It is increasingly recognized that apoptosis of MSCs is a critical component of their therapeutic efficacy [114]. Direct application of apoptotic bodies derived from MSCs promotes wound healing and M2 macrophage polarization [84]. Additionally, macrophages preconditioned with MSC apoptotic bodies secrete paracrine factors that promote fibroblast migration and proliferation [84]. 

Toll-like receptors (TLRs) are a key component of the innate immune system that recognizes pathogen-associated molecules. While TLRs are important in the acute phase for the clearance of pathogens, their sustained activity can be maladaptive [115]. Chronic venous leg ulcers have higher levels of TLR-2 and TLR-4 [116]. MSC-EVs can modulate macrophage reactivity to LPS (TLR-4 ligand) by transfer of miR-let7b [94] and miR-181c [101], resulting in attenuated TNF-α and IL-1β production and stimulating the production of anti-inflammatory TGF-β and IL-10 [94,101].

Progressive mitochondrial dysfunction is associated with aging and chronic inflammation [117], which contributes to chronic wound formation [118]. An intriguing additional mechanism for promoting M2 polarization is through the transfer of mitochondria [119]. Inflammatory M1 macrophages rely on glycolysis, whereas the anti-inflammatory M2 phenotype is more dependent on mitochondrial oxidative phosphorylation [120]. Additionally, in a murine model of acute oxidative stress, MSC-EVs can reduce ROS-associated skin inflammation in response to ultraviolet irradiation and protect mitochondria from oxidative stress [121]. 

T-lymphocyte recruitment occurs late in the inflammatory phase. Regulatory T-cells (T_regs_) function to limit inflammation, thereby protecting viable cells from immune-mediated damage. T_regs_ promote neutrophils secretion of anti-inflammatory molecules and promote neutrophil apoptosis. They also can polarize macrophages towards the M2 phenotype [122]. Amphiregulin is an EGF-like growth factor that can induce the local release of bio-active TGF-β. Tissue resident T_regs_ have been proposed to maintain an environment conducive for proper wound healing through this localized amphiregulin/TGF-β cascade [123]. Tissue resident γδT-cells secrete keratinocyte growth factors and IGF-1 to promote keratinocyte proliferation and survival [124]. Mice deficient in B-cells and T-cells have been shown to have scar-free healing [125]. Furthermore, depletion of T-cells impairs collagen deposition and decreases wound strength [126]. These findings indicate an important role for T-cells in the proliferation and remodeling phases.

Dendritic cells (DCs) are the primary antigen presenting cell of the immune system and are a key link between the innate and adaptive immune responses. MSC-EVs impair DC antigen uptake and expression of co-stimulatory molecules [127]. DC treatment with MSC-EVs reduced the secretion of IL-6 and IL-12p70 inflammatory cytokines, reduced the expression of CCR7 chemokine receptor, and increased secretion of anti-inflammatory TGF-β. These effects were attributed to EV-mediated transfer of miRNAs, in particular miR-21-5p [127]. Through their action on DCs, MSC-EVs are able to attenuate the production of inflammatory T-cells and shift production towards FOXP3^+^ regulatory T-cells [128,129]. MSC-EVs were also shown to inhibit inflammatory T-cell differentiation, proliferation, activation, and IFN-γ production [130].

The inflammatory response in cutaneous wound healing must remain in homeostasis. The initial burst of inflammation is critical for clearing pathogens and debris. Then the inflammation must resolve to make way for the next phases of the healing process. An excessive inflammatory response will damage surrounding healthy tissues and a prolonged response will delay wound closure. MSC-EVs display promising immunomodulatory effects for promoting an inflammatory environment conducive to effective wound healing. 

### 3.2. Proliferation

The proliferative phase involves creating a new foundation upon which the epithelial barrier will rest. In the dermis, this involves angiogenesis, fibroblast proliferation, and provisional ECM deposition to create granulation tissue. The wound environment is metabolically active and requires new blood vessel formation to supply these demands. Failure to supply adequate metabolic nutrients can delay or disrupt the healing process [131]. Additionally, the high glucose environment of diabetes mellitus can inhibit endothelial cell and fibroblast proliferation and promotes their apoptosis [132] (Figure 2b). 

MSC-EVs stimulate the expression of repair associated growth factors that promote neoangiogenesis in murine wound models (Table 1). In vitro, MSC-EVs can promote endothelial cell proliferation, migration, tube formation, and secretion of VEGF [81,98,100]. It was demonstrated that MSC-EVs stimulate the AKT/eNOS pathway to promote angiogenesis, in part through the transfer of miR-221-3p [98]. Transfer of miR-31, miR-125a, miR-126, and circRNA mmu_circ_0000250 have also be shown to support endothelial cell proliferation and tube formation [91,96,133,134]. Endothelial progenitor cells cultured in high glucose conditions undergo premature senescence. MSC-EVs can protect endothelial progenitor cells from senescence by inhibiting the expression of inflammatory cytokines and limiting ROS production [95]. 

MSC-EVs also stimulate fibroblast proliferation, migration, and ECM production in vivo (Table 1). MSC-EVs have been shown to carry EGF, FGF2, Wnt3a, and Wnt4, which can be delivered to dermal fibroblasts, stimulating their migration and collagen synthesis [67,135,136,137]. Cultured fibroblasts treated with MSC-EVs increase the expression of growth factors (EGF, FGF2, VEGF, PDGF) and ECM molecules (Fibronectin, Collagen 1, Collagen III, Elastin) [81,138]. The function of fibroblasts derived from chronic wounds can be enhanced by treatment with MSC-EVs in a dose-dependent manner, which may be mediated by EV transfer of STAT3 [139]. MSC-EVs can also stimulate AKT and ERK signaling in fibroblasts which have been correlated with enhanced repair functions [79,81]. 

Finally, MSC-EVs accelerate wound re-epithelialization (Table 1). Keratinocytes treated with MSC-EVs in vitro display enhanced proliferation and migration [138], accompanied by increased expression of VEGF, fibronectin, c-MYC, and MMP-9 [81]. MSC-EVs were shown to accelerate re-epithelialization via transfer of miR-27b, leading to activation of JUNB/IRE1α signaling [82]. Additionally, MSC-EVs can promote re-epithelialization and keratinocyte proliferation through AKT/HIF-1α signaling [86]. MSC-EVs can also protect keratinocytes from oxidative stress-induced apoptosis by inhibiting nuclear translocation of AIF and suppressing activation of PARP1 [87]. 

MSC-EVs may also promote repair through stimulation of tissue resident stem cells, though less is known if this occurs in cutaneous wound healing. MSC-EVs can increase the stemness of human dermal fibroblasts through the transfer of OCT4 and NANOG [140]. BM-MSCs and MSC-EVs undergo an age-related decline in reparative capacities [141]. It was shown that MSCs from aged rats expressed lower levels of pluripotency markers OCT4 and NANOG [142]. Incubation of old MSCs with MSC-EVs from young rats increased expression of OCT4 and NANOG and decreased expression the senescence marker Vinculin [142]. Additionally, it was shown that EVs from young MSCs can delay premature senescence, improve stemness, and stimulate glycolytic metabolism in old MSCs [143]. Finally, MSC-EVs can promote tendon repair by suppressing apoptosis of tendon stem cells [144]. Additional studies will be needed to determine how MSC-EVs influence cutaneous stem cell populations. 

### 3.3. Remodeling

The remodeling phase is critical for strengthening the repaired wound. In this phase the provisional ECM is replaced with thicker and more organized collagen bundles, resulting in an increase in tensile strength over a period of months [102]. The wound will also contract, which is mediated by myofibroblasts. If any phase of the healing process is disrupted, atrophic scars, hypertrophic scars, keloids, and chronic wounds can result. 

MSC-EVs can decrease fibroblast collagen deposition, the trans-differentiation of fibroblasts to myofibroblasts, and the formation of hypertrophic scars [88]. MSC-EVs were found to express miR-192-5p, which suppresses the pro-fibrotic IL-17RA/SMAD axis [88]. TSG-6 is a secreted glycoprotein with anti-inflammatory effects and is noticeably reduced in keloid scars [145]. MSC-EVs contain TSG-6 protein and in an in vivo model MSC-EVs limited scar formation in a TSG-6 dependent fashion [83]. TSG-6 delivery resulted in reduced expression of TGF-β1, Collagen I/III, and phosphorylated-SMAD2/3 [83]. MSC-EVs are also enriched in several miRNAs (miR-21, -23a, -125b, and -145) that can inhibit TGF-β/SMAD2 signaling and suppress myofibroblast formation [77]. 

The effect of MSC-EVs on fibroblasts has been reported to either increase or decrease function between studies or within a study at different time points. One potential explanation for how this paradoxical effect may occur is through the generation of regulatory macrophages. Regulatory macrophages are anti-inflammatory and anti-fibrotic, whereas M2 macrophages are pro-fibrotic [146]. While MSC-EVs can enhance the anti-inflammatory phenotype of regulatory polarized macrophages [147], it is unknown if MSC-EVs enhance the anti-fibrotic effects. 

## 4. Tailoring EVs to Heal Chronic Wounds

Pre-clinical work has demonstrated great promise for the use of MSC-EVs for treating chronic wounds. Numerous studies have found ways to further enhance the wound healing efficacy of EVs, which will be discussed in the following sections. As we learn more about the pathophysiology of chronic wounds, it can be envisioned that MSC-EVs can be personalized to an individual patient based on wound etiology, co-morbidities, and any underlying biological defect in the wound healing process. 

### 4.1. Extracellular Vesicles: Source

As previously noted, MSCs are known to be a highly heterogeneous population, and unsurprising, EVs derived from MSCs also show significant variability. EV production is influenced by the source cell, passage number, growth media, atmosphere, culture substrate, and collection conditions. Successful clinical implementation of EVs will also require a means to produce enough EVs. Fortunately, MSCs are one of the most active producers of EVs [148]. EV production can be enhanced by various stimuli, such as hypoxia [149], low pH [150], 3D cell culture [151], acoustic-, electrical-, and mechanical-stimulation [152,153,154,155]. Methods for enhancing intrinsic MSC production of EVs have been reviewed elsewhere [156]. Given the prevalence of chronic wounds, economical large-scale production methods will be needed to generate MSC-EVs for this to be a broadly applicable therapy. Standard cell culture vessels are inefficient for large-scale MSC-EV production. Bioreactor systems provide a scalable system for generating large quantities of clinical-grade EVs [157,158]. 

MSC-EV cargo and downstream effects vary depending on where MSCs are harvested from. With regard to wound healing, Hoang et al. evaluated how MSC source influences EV function. They found that BM-MSC-EVs contained the highest levels of FGF2 and PDGF-BB and displayed the strongest effect on fibroblasts. Whereas, UC-MSCs contained the highest levels of TGF-β and produced the greatest effect on keratinocytes [135]. Comparative analysis of BM-MSC-EV and AD-MSC-EV content revealed that both types are enriched in miRNAs targeting EGF, PI3K/AKT, TGF-β signaling pathways [93]. AD-MSC-EVs are enriched in proangiogenic miRNAs that target HIF-1 and other angiogenic proteins (TGF-β, FGF, PDGFR, TNF, ANGPT1). BM-MSC-EVs contained more abundant proteins linked to integrin and cadherin signaling and metabolic processes [93]. 

Production of EVs by MSCs is also age-dependent. MSCs from older individuals and late-passage cultures produce more EVs [159,160]. Importantly, these EVs have different cargos and may not produce the desired therapeutic effects [161,162]. Qui et al. showed that adult BM-MSCs pre-treated with neonatal serum EVs have enhanced wound healing potential. Furthermore, these “rejuvenated” BM-MSCs secreted EVs that are superior at promoting wound healing, inducing endothelial cell proliferation, and stimulating AKT/eNOS signaling [85]. Comparison of MSC-EVs from young and aged mice identified enrichment of miR-126 in young MSC-EVs [163]. Overexpression of miR-126 in aged MSCs, results in the production of EVs with potent angiogenic potential, equivalent to EVs from young MSCs [163]. These findings have implications when designing therapies for chronic wounds. MSCs would ideally be harvested from younger donors and MSCs would not be expanded beyond an early number of passages. When this is not feasible, it may be possible to use young MSC-EVs or molecules to “rejuvenate” sub-optimal MSCs to produce EVs with better biologic activity. 

Environmental stimuli also influence MSC-EV characteristics. Growing MSCs in a hypoxic atmosphere or the use of hypoxia-mimetic molecules increases EV yield and increases the angiogenic potential of isolated EVs [164,165,166,167,168]. Hypoxia increases VEGF, EGF, FGF, VEGF-R2, VEGF-R3, MCP-2, and MCP-4 in AD-MSC-EVs, which correlates with more robust angiogenic potential [167]. EVs from MSCs treated with dimethyloxaloylglycine stimulate angiogenesis by activating AKT/mTOR signaling [168]. The hypoxia-mimetic deferoxamine when added to BM-MSCs results in the production of EVs with increased wound healing and pro-angiogenic properties [96]. It was shown that in part this was through EV delivery of miR-126 to recipient cells, resulting in PTEN suppression [96]. 

Inflammation stimulates MSCs to generate immunosuppressive EVs [169]. EVs from MSCs stimulated with TNF-α and IFN-γ promote M2 macrophage polarization, potentially through changes in miRNA content, resulting in IRAK1 inhibition [170]. Additionally, MSCs preconditioned with TNF-α and IFN-γ generate EVs with elevated COX2, leading to the generation of anti-inflammatory PGE_2_ [171]_._ Ti et al. showed, in a diabetic wound model, that EVs from LPS preconditioned MSCs decreased inflammatory cell infiltration into the wound and polarized macrophages towards the M2 phenotype. LPS preconditioned MSC-EVs were enriched with let-7b, miR-1180, miR-183, miR-550b, and miR-133a. Transfer of let-7b to macrophages leads to M2 polarization through inhibition of TLR4/NF-kB and stimulation of STAT3 and AKT signaling [94]. 

The culture substrate is another modifiable factor when generating tailored MSC-EVs [172]. MSCs grown on a fibrous scaffold or as spheroids enhance their secretion of paracrine mediators that promote wound healing [173,174]. Growing MSCs in 3D culture enhances the secretion of galectin-1, promoting the proliferation and migration of keratinocytes and fibroblasts [175]. The role of EVs in these studies was not specifically addressed, but EVs would have been present in the MSC conditioned media based on the methods reported. A recent study found that 3D culture of UC-MSCs generates EVs that promote fibroblast proliferation and migration [176]. 

Based on the preceding findings, when MSCs are stimulated by factors found in the chronic wound environment they produce EVs with more potent wound healing potential. When MSCs are exposed to hypoxia, they generate EVs that promote angiogenesis, and when they are exposed to inflammatory molecules, they produce immunomodulatory EVs. These observations are congruent with MSCs, and by extension with MSC-EVs, being critical regulators of tissue homeostasis. It should be explored if a combination of environmental factors can further enhance the bioactivity of MSC-EVs for chronic wound applications. 

The cargo of MSC-EVs can also be influenced by targeting MSC receptors. Melatonin promotes MSCs to produce EVs with enhanced anti-inflammatory and wound healing activity [97]. Melatonin MSC-EVs enhance wound closure, Collagen I and III expression, and M2 macrophage polarization compared to untreated MSC-EVs. Melatonin MSC-EVs attenuate inflammation by suppressing AKT signaling [97]. EVs collected from atorvastatin treated MSCs display enhanced angiogenic effects, mediated by miR-221-3p upregulation and AKT/eNOS activation in endothelial cells [98]. It is intriguing to note that MSCs express light-sensing proteins that are typically expressed by retinal photoreceptors. MSCs stimulated with blue (455 nm) light released EVs with more potent angiogenic potential [177]. Blue light stimulation was noted to increase miR-135b and miR-499a packaging into EVs [177].

Multiple techniques exist for isolating EVs including ultracentrifugation (differential, density-gradient, and sucrose cushion), size-exclusion chromatography, immunoaffinity, microfluidics, and others [178]. The advantages and disadvantages of each technique have been reviewed elsewhere [179,180]. For example, Wnt3a is bound to the exterior of BM-MSC-EVs. Traditional ultracentrifugation dislodges Wnt3a, but a combination of polyethylene-glycol enrichment with sucrose cushion ultracentrifugation allows for the recovery of EVs with bound Wnt3a [67]. The type of isolation method employed must consider cost, safety, and the quantity, quality, and biologic-activity of recovered EVs. 

### 4.2. Extracellular Vesicles: Engineering

There is tremendous interest in selectively engineering EVs to maximize their delivery of bioactive molecules and to target them to specific cell populations [181,182,183,184,185,186,187]. The surface of EVs can be modified for display of therapeutic molecules or modulate cell targeting. MSCs can be genetically engineered to display peptide sequences, proteins, and antibody fragments fused to the extracellular domain of EV transmembrane proteins. The exterior can be further modified post-isolation by conjugating molecules to surface proteins (e.g., “click” chemistry) and insertion of amphipathic molecules into the lipid bilayer [187]. Modification of the EV surface has been largely unexplored in wound healing research, but it has the potential for substantial therapeutic benefit. For example, it may be possible to insert palmitoylated proteins such as Wnt proteins into isolated EVs. 

The most frequently employed method for enriching EV cargo is to overexpress the coding DNA sequence in the EV source cells. This technique has been successfully utilized in wound healing studies. The transcription factor NRF2 provides protection against oxidative stress in diabetic models. EVs derived from NRF2 overexpressing AD-MSCs, compared to standard AD-MSC-EVs, promote faster wound healing in vivo and protect cultured endothelial progenitors from senescence by inhibiting ROS and inflammatory cytokines [95]. MSC-EVs loaded with lncRNA H19 can modulate the miR-152-3p/PTEN axis in fibroblasts grown from diabetic foot ulcers [90]. These H19 loaded MSC-EVs promoted wound healing in a mouse diabetic wound model, suppressed inflammation, and decreased apoptosis [90]. MSC-EVs enriched with TSG-6 showed superior ability to reduce scar formation compared to standard MSC-EVs [83]. 

Other methods have been developed to target proteins to EVs that are not normally loaded into EVs. The ‘exosomes for protein loading via optically reversible protein–protein interactions’ (EXPLORs) technique uses a light reversible linker to attach proteins to CD9, an EV associated tetraspanin molecule [188]. Another technique proposed is to capture proteins in self-assembling structures such as ‘enveloped protein nanocages’ [189]. 

Additional methods have been proposed to induce EV formation while bypassing active cargo sorting mechanisms, thereby producing EVs with a sampling of all cytoplasmic molecules. EVs generated by these means are also referred to as extracellular vesicle mimetics or cell-engineered nanovesicles [190,191]. Vesicle production can be induced by subjecting cells to hypotonic solution followed by osmotic vesiculation buffer [192]. Cytochalasin B is a pharmacologic agent that disorganizes the actin cytoskeleton. When cytochalasin B treated MSCs are then subjected to shearing stress (vortexing) they produce immunomodulatory and angiogenic EVs [193,194]. EVs can also be generated by extruding cells through 1 μm- or 2 μm-pore polymer filters [195], or by ultrasonication [196]. 

Isolated EVs can be passively loaded with drugs that can pass through the lipid bilayer, whereas other molecules need additional assistance to enter EVs. Active methods such as electroporation, sonification, freeze/thaw, extrusion, saponin, and transfection reagents can allow additional cargos into EVs [183]. Most methods discussed are inefficient at incorporating large molecules into EVs. Engineered lipid nanoparticles can be loaded with high concentrations of therapeutic molecules, but have inferior biocompatibility compared to EVs [197]. Hybrid exosome-liposome vesicles can be generated through co-incubation, freeze-thaw, and sonication [197]. These hybrid vesicles possess the membrane proteins important for EV biodistribution and targeting while incorporating large molecules into the vesicle [198]. 

### 4.3. Extracellular Vesicles: Quality Control

Rigorous quality control metrics must be established prior to the clinical application of EVs [199]. Each batch of EVs needs to be assessed for its identity, purity, and potency to ensure safety and therapeutic efficacy. Multiple assays will be necessary to fully evaluate EVs given their complex biology. The identity and purity of a batch can be evaluated by measuring the ratio of MSC to non-MSC EV surface antigens and size distribution [199]. The quantity of any specific therapeutic molecules should also be assessed between batches. 

One of the challenges with a clinical translation of EVs is optimal dosing. Many studies do not include a dose–response curve to optimize the proper concentration for efficacy. The question remains to be determined if higher dosing results in better/faster healing or if there is a plateau or negative effect from overdosing. MSC-CM and MSC-EVs have been shown to stimulate wound healing responses in a dose-dependent manner, though there is a ceiling to their effect [139,200]. 

Furthermore, the potency of an EV preparation must be determined to provide a consistent therapeutic dose. Most studies report EV dose as either the number of vesicles or protein content delivered. It would be more appropriate to calculate dose as biologically active “units” based on functional assays. Potency testing for wound healing could involve any combination of in vivo wound healing assays in model species, or in vitro assays to measure their effect on keratinocytes, fibroblasts, endothelial cells, and immune cells. An in depth discussion of functional assays for EVs can be found elsewhere [201]. 

### 4.4. Extracellular Vesicles: Delivery

Cutaneous wounds provide multiple options for MSC-EV delivery. Most murine studies injected EVs locally near the wound (Table 1). This method is not ideal clinically as it could cause significant pain and distress to the patient. Intravenous injection provides an alternative if the patient has multiple wounds, or a large body surface area is involved. Intravenous (IV) injection of MSC-EVs tagged with iron oxide nanoparticles can be directed to an injury site with a magnet [202]. Hu et al., demonstrated that IV injection of fluorescently tagged MSC-EVs in a mouse wound model showed fluorescence in the wound site on days 5–14 following injury and MSC-EV injection [78]. Their study showed that initially the MSC-EV fluorescence signal was restricted to the spleen on day 1 and then fluorescence accumulated in the injury site days later [78]. When considering the short circulatory half-life of EVs (minutes to hours, see Section 2.2), it is difficult to explain how EVs remain in circulation long enough to correlate with these findings. It may be that EVs rapidly accumulate in the spleen and then are slowly released back into circulation. Alternatively, EVs may act on splenic cells that are then released in response to inflammatory cues [203]. Further work will be needed to evaluate which cells in the wound environment are targets of MSC-EVs. This question could be addressed by identifying which cells in the wound accumulate tracer carried by EVs, though the signal may not reach the limit of detection with this method. An alternative would be to use EVs loaded with molecules capable of inducing stable changes in recipient cells (Cre recombinase or CRISPR/Cas9) [204]. 

Topical application is appealing because it minimizes patient discomfort, enables a high dose of EVs to be delivered directly to the wound, and allows EVs to be delivered as biomatrices to further enhance wound healing. Topical application of MSC-EVs loaded into carboxymethylcellulose, alginate, or Pluronic F127 hydrogels promote wound healing and neoangiogenesis [89,93,99]. EVs can also be loaded onto hydrogels designed with anti-microbial and adhesive properties suited for the wound environment [89]. Work done in our laboratories has shown that EVs can be stabilized in a collagen scaffold without diminution in efficacy for anti-inflammatory therapies in an osteoarthritis model as well as provide sustained release up to one week in vitro [205](and unpublished observations, D.A.G.). Wang et al., demonstrated that a complex hydrogel system (FHE—Pluronic F127, hyaluronic acid, and poly-ε-l-lysine) could provide pH-responsive sustained EV release and promote skin repair in a diabetic wound healing model [89]. 

## 5. Clinical Perspectives

Stem cell products including EVs are regulated and require FDA approval. Currently, the only stem cell products that are FDA-approved for use in the United States consist of hematopoietic progenitor cells that are derived from umbilical cord blood for use in patients with disorders that affect the production of blood.

There are currently no FDA-approved EV products (https://www.fda.gov/vaccines-blood-biologics/consumers-biologics/consumer-alert-regenerative-medicine-products-including-stem-cells-and-exosomes, accessed on 16 September 2021). At the time of this publication, clinicaltrials.gov (accessed on 16 September 2021) listed three trials using EVs to treat chronic wounds. Two clinical trials will evaluate if serum derived EVs can induce a change in wound size and associated pain (NCT02565264 and NCT04652531). 

One clinical trial (NCT04173650) will evaluate MSC-EV dose-limiting toxicity and wound size in dystrophic epidermolysis bullosa. Recessive dystrophic epidermolysis bullosa is an inherited skin fragility disorder, due to mutations in the COL7A1 gene, resulting in defective anchoring of the epidermis to the dermis [206]. Affected children suffer from generalized skin blistering, ulceration, and scarring, for which there is no definitive cure. BM transplant and MSC treatment can increase Collagen VII in the skin [207,208,209]. Work done in our laboratories demonstrated that MSC-EVs are capable of transferring Collagen VII mRNA and protein to fibroblasts [210]. Additionally, EVs may provide an ability to rejuvenate skin cell damage [211].

MSC-EVs can also be considered for an adjuvant role to other modalities. Skin flaps and grafts are part of the clinical toolkit for treating wounds, but flap/graft failure is a major clinic challenge and can prolong the course of a chronic wound. In an in vivo model of flap ischemia-reperfusion injury, EVs increased the rate of flap survival, reduced inflammatory cell infiltrate, and induced neoangiogenesis [164]. Additionally, MSCs were shown to delay the rejection of MHC-mismatched skin grafts in immunocompetent baboons [212]. These findings indicate that MSC-EVs could be an adjuvant therapy when using allografts to reduce immune-mediated graft rejection.

MSC-EVs provide many benefits relative to their parent cell. MSC-EVs are more stable than MSCs. Unlike MSCs, experiments monitoring EV biodistribution have not reported significant pulmonary accumulation. Transplantation of genetically engineered MSCs carries a risk for tumorigenesis and ectopic tissue formation should they become stably incorporated into the host. EVs carry a finite quantity of bioactive molecules; thus, mitigating the risk. A wide array of modifications can be applied to EVs to enhance their intended therapeutic purpose. Limitations to MSC-EV therapeutics include scarcity of the source cell should BM-MSCs be used, limited yield of EVs per production batch, heterogeneity among EVs, and lack of standardized quality control and potency assays. All cell-based therapies have the potential to transmit infectious diseases. While most infectious diseases can be screened for, no approved method for detecting prions has been approved. Human platelet lysate appears to be an alternative to bovine serum in MSC culture, with MSC-EVs showing comparable immunomodulatory effects [213]. 

The International Society of Extracellular Vesicles has published a position paper outlining important considerations regarding the application of EVs in clinical trials [214]. The clinical application of MSC-EVs in wound healing will require the development of manufacturing strategies compliant with good manufacturing practices (GMP). Additionally, robust quality control and potency testing will be needed to fulfill regulatory requirements. 

## 6. Conclusions

Pre-clinical data indicate that MSC-EVs can accelerate wound healing by modulating the immune response and by promoting angiogenesis, fibroblast function, and re-epithelialization. There are numerous methods available to modify the cargo of EVs, making them a versatile drug delivery system. MSC-EVs can be delivered intravenously, injected into the wound site, or applied topically to treat chronic wounds. This flexibility in the design and delivery of MSC-EVs opens the doors for creating personalized therapies for chronic wounds. 

## Abbreviations

AIF	Apoptosis-inducing factor
CCR7	C-C motif chemokine receptor 7
COL7A1	Collagen VII alpha 1 chain
CM	Conditioned media
DC	Dendritic cell
ECM	Extracellular matrix
EGF	Epithelial growth factor
eNOS	Endothelial NOS
ESCRT	Endosomal sorting complex required for transport
EV	Extracellular vesicle
FHE	Pluronic F127, hyaluronic acid, and poly-[epsilon]-L-lysine
FGF	Fibroblast growth factor
FOXP3	Forkhead box P3
HIF-1α	Hypoxia inducible factor-1-alpha
IL	Interleukin
IL-1RA	Interleukin 1 receptor antagonist
IL-17RA	Interleukin 17 receptor A
IFN	Interferon
IGF-1	Insulin-like growth factor 1
IRE1α	Inositol requiring enzyme-1-alpha
IV	Intravenous
kDa	kilodalton
LPS	Lipopolysaccharide
MMP-9	Matrix metalloproteinase 9
NRF2	Nuclear factor erythroid 2 like 2
OCT4	Octamer-binding protein 4
PARP1	Poly (ADP-ribose) polymerase-1
PDGF	Platelet-derived growth factor
PTEN	Phosphate and Tensin homolog
ROS	Reactive oxygen species
STAT3	Signal transducer and activator of transcription 3
SIRPα	Signal regulatory protein alpha
TGF-β	Tissue growth factor beta
THBS1	Thrombospondin 1
TLR	Toll-like receptor
TNF-α	Tumor necrosis factor alpha
T_regs_	Regulatory T lymphocyte
TSG-6	Tumor necrosis factor-stimulated gene-6
VEGF	Vascular endothelial growth factor
MSC	Mesenchymal stem cell
BM-MSC	Bone marrow MSC
AD-MSC	Adipose tissue MSC
UC-MSC	Umbilical cord MSC
mRNA	Messenger RNA
mtDNA	Mitochondrial DNA
rRNA	Ribosomal RNA
tRNA	Transfer RNA
lncRNA	Long noncoding RNA
circRNA	Circular RNA
piRNA	picoRNA
